# Unaddressed participants’ gaze in multi-person interaction: optimizing recipiency

**DOI:** 10.3389/fpsyg.2015.00098

**Published:** 2015-02-09

**Authors:** Judith Holler, Kobin H. Kendrick

**Affiliations:** Language and Cognition Department, Max Planck Institute for PsycholinguisticsNijmegen, Netherlands

**Keywords:** turn-taking, turn projection, eye gaze, eye-tracking, unaddressed participants

## Abstract

One of the most intriguing aspects of human communication is its turn-taking system. It requires the ability to process on-going turns at talk while planning the next, and to launch this next turn without considerable overlap or delay. Recent research has investigated the eye movements of observers of dialogs to gain insight into how we process turns at talk. More specifically, this research has focused on the extent to which we are able to anticipate the end of current and the beginning of next turns. At the same time, there has been a call for shifting experimental paradigms exploring social-cognitive processes away from passive observation toward on-line processing. Here, we present research that responds to this call by situating state-of-the-art technology for tracking interlocutors’ eye movements within spontaneous, face-to-face conversation. Each conversation involved three native speakers of English. The analysis focused on question–response sequences involving just two of those participants, thus rendering the third momentarily unaddressed. Temporal analyses of the unaddressed participants’ gaze shifts from current to next speaker revealed that unaddressed participants are able to anticipate next turns, and moreover, that they often shift their gaze toward the next speaker before the current turn ends. However, an analysis of the complex structure of turns at talk revealed that the planning of these gaze shifts virtually coincides with the points at which the turns first become recognizable as possibly complete. We argue that the timing of these eye movements is governed by an organizational principle whereby unaddressed participants shift their gaze at a point that appears interactionally most optimal: It provides unaddressed participants with access to much of the visual, bodily behavior that accompanies both the current speaker’s *and* the next speaker’s turn, and it allows them to display recipiency with regard to both speakers’ turns.

## INTRODUCTION

The contrast formed by the white sclera surrounding a darker iris and pupil is unique to the human eye (Kobayashi and Kohshima, 2001). This contrast renders eye gaze a highly salient cue in interaction with others, and the pivotal role gaze plays in human communication has been demonstrated by numerous studies (see Argyle and Cook, 1976; Cook, 1977; Kleinke, 1986; Itier and Batty, 2009; Senju and Johnson, 2009; Rossano, 2012 for reviews). By now, we know a great deal about how gaze functions in dyadic encounters, such as to initiate interaction, signal address, receive addressee feedback, and coordinate turn transitions (e.g., Kendon, 1967, 1990; Argyle et al., 1973; Cary, 1978; Duncan et al., 1979; Goodwin, 1980; Bavelas et al., 2002; Lerner, 2003; Rossano et al., 2009). Here, we study gaze behavior with respect to another core aspect of social interaction, namely the precise timing of gaze and turns at talk in multi-person interaction. More precisely, we investigate how the cognitive processing of turns infuences gaze behavior of momentarily unaddressed participants during question–response sequences and consider the social opportunities this may create in a triadic conversation context.

### THE TIMING OF TURNS AT TALK

In social interaction, a system of turn-taking organizes opportunities to speak. According to Sacks et al. (1974), turns at talk are constructed out of linguistic units that have recognizable structures, enabling a next speaker to project the structure in advance and, consequently, anticipate the possible completion of the unit. Subsequent research has examined the syntactic and prosodic structures that allow for the projection of a current turn and signal its possible completion (Ford and Thompson, 1996; Ford et al., 1996; Selting, 1996; Wells and Macfarlane, 1998; Auer, 2005; Local and Walker, 2012). Within the model, the first possible completion of such a unit constitutes a place, referred to as a transition-relevance place, at which a transition from current to next speaker may occur (Sacks et al., 1974; Selting, 2000). A set of rules and constraints in the model, such as a constraint on more than one speaker at a time (Sacks et al., 1974; Jefferson, 1986; Schegloff, 2000), accounts for the observation that transitions tend to occur with minimal overlap between turns. At the same time, rules, and constraints in the model lead to minimal gaps between turns. This is particularly remarkable since quantitative studies have shown that gaps between turns are most frequently on the order of just 0–200 ms (Stivers et al., 2009; Heldner and Edlund, 2010). As Levinson (2013) has argued, short gaps between turns do not provide adequate time to prepare even a simple next turn, which psycholinguistic research has shown requires at least 600 ms (Indefrey and Levelt, 2004; Indefrey, 2011). This suggests that a next speaker must begin to plan the next turn well before the current one is complete, a psycholinguistic challenge in which projection of a current turn appears to play an important role (De Ruiter et al., 2006; Magyari and de Ruiter, 2012; Magyari et al., 2014).

### THIRD-PERSON PERSPECTIVE EYE-TRACKING STUDIES ON TURN-TAKING

Recently, a new experimental paradigm has been developed for the study of the cognitive processes that underpin turn-taking from a third-person perspective. The general procedure involves participants being presented with a pre-recorded dialog or conversation between two people on a computer screen while their eye movements are tracked and timed with respect to the turns at talk they hear. Experimental studies using this novel paradigm have shed light on the precise timing of eye movements and turns at talk by measuring where observers of dialogs look and when they do so.

A study by Augusti et al. (2010) has shown that infants of just 6 months of age shift their gaze from current speaker to next speaker in accordance with the alternation of turns, thus, they argue, showing a sensitivity to the natural flow of conversation. Other studies have shown that, at least by 3 years of age, children are not only able to track who is speaking at any one time, but they are indeed able to anticipate upcoming turns, shifting their gaze to the next speaker often *before* he or she begins to speak (von Hofsten et al., 2009; Casillas and Frank, 2012, 2013; Keitel et al., 2013).

Studies using the same paradigm with adults have shown that they, too, tend to look reliably at the current speaker (Tice and Henetz, 2011; Casillas and Frank, 2012; Edlund et al., 2012; Hirvenkari et al., 2013). However, these studies have yielded discrepant findings regarding when observers begin to look to the next speaker. Foulsham et al. (2010) asked observers to watch a video of others performing a conversation-based group-decision task and to decide whom of these they would like to work with on a subsequent task. Their findings showed that observers fixated the next speaker on average 150 ms before they started to speak. Tice and Henetz (2011), Casillas and Frank (2012), and Keitel et al. (2013) measured the eye movements of observers of dialogs. Keitel et al. (2013) found that 54% of adults’ gaze shifts occurred within a time window starting 500 ms prior to the end of the current turn and ending with the beginning of the next turn. The gaze shifts thus occurred while the current speaker was still talking, or during the gap between turns, providing clear evidence of anticipation of the next turn. Similarly, Tice and Henetz (2011) and Casillas and Frank (2012) found that the majority of their participants’ eye movements to the next speaker occurred either during the gap between turns or within the first 200 ms of the next turn. Since it takes around 200 ms for a saccadic eye movement to be planned and launched (Salthouse and Ellis, 1980; Fischer and Ramsperger, 1984; Becker, 1991; Allopenna et al., 1998; Griffin and Bock, 2000), these gaze shifts must have been planned *prior* to the beginning of this next turn. Moreover, in at least some cases, observers shifted their gaze to the next speaker even before the *current* turn had ended (Casillas and Frank, 2012, 2013). Together, the findings from these studies suggest that observers of scripted dialogs and spontaneous group conversations engage in predictive cognitive processes that allow them to anticipate the beginnings of next turns, and, at least to some extent, also the ends of current turns.

However, two studies using truly spontaneous (rather than scripted or performed) dialogs have not found evidence for anticipatory looks to the next speaker. Edlund et al. (2012), too, have shown that observers track current speakers with their gaze, and although the precise timing of this gaze with respect to turn transitions is not provided, the data they do provide seem to suggest that looks to the next speaker before he or she started to speak were rare, if present at all. Hirvenkari et al. (2013), too, found that their observers looked at the next speaker only after he or she had already begun to speak. One possible reason for this, they state, could be that participants in other studies (e.g., Foulsham et al., 2010) may have been more eager to see the reactions of the participants due to the decision task they were asked to complete. They argue that the gaze behavior of their participants merely observing dialogs may have been “less tightly linked to the turn-taking than if the task would have been more engaging, or if the subjects would have actually taken part in the conversation” (Hirvenkari et al., 2013, p. 6). Thus, it is evident that the nature of the experimental task and the spontaneity of the conversational exchange may influence the temporal coupling of observers’ eye movements and turns at talk. An investigation of the timing of eye movements and speaking turns while participants are engaged in actual conversation, processing spontaneous turns without them being required to complete an experimental task, is therefore an important next step.

### METHODOLOGICAL CONSIDERATIONS

While there is some discrepancy in findings, studies using the novel third-person perspective eye-tracking paradigm described above have provided us with valuable first insights into how adults may process turns at talk and transitions between them, as well as how children acquire this skill during development. However, two issues emerge from this work.

The first issue has already been alluded to in the preceding section and concerns the third-person perspective as such. Recently, Schilbach (2010, 2014) and Schilbach et al. (2013) put forward a convincing argument for the urgency of a shift in experimental paradigm, stating that “recent conceptual and empirical developments consistently indicate the need for investigations that allow the study of real-time social encounters in a truly interactive manner. This suggestion is based on the premise that social cognition is fundamentally different when we are in interaction with others rather than merely observing them” (Schilbach et al., 2013, p. 393). Their argument, and the evidence they cite, concerns the abundance of paradigms in the field of cognitive neuroscience involving passive observation and the different insights interactive paradigms have provided in this domain. The latter immerse participants in ‘online’ social interaction rather than ask them to observe oﬄine interactions, thus creating reciprocal relations with sequences of actions and reactions shaping the communication between the participants (Wilms et al., 2010; Pfeiffer et al., 2013).

One important question that remains, therefore, is when participants shift their gaze from current to next speaker if they themselves are ratified participants in the conversation but momentarily unaddressed (Goffman, 1979, 1981; Clark and Carlson, 1982). If the degree of engagement that participants feel indeed influences their ability (or motivation) to project either current or next turns, then we might see more evidence of early gaze shifts when participants are directly immersed in a live conversation. An alternative possibility is, however, that the considerably reduced social context of third-person perspective paradigms underestimates the cognitive demands placed on processing turns at talk in spontaneous conversation. Participants may thus have *less* cognitive resources available for projection in live conversation, meaning gaze shifts may be primarily responsive to the next speaker beginning to speak rather than anticipatory. However, it could also be that eye movements in face-to-face interaction do not reflect the projection of current or next turns at all, but that the social norms and dynamics of conversation determine where participants look and when. Thus, while third-person perspective eye-tracking paradigms allow for a high degree of experimental control and manipulation to investigate eye movements during turn-taking, and the role semantics and prosody play in this context (Casillas and Frank, 2013; Keitel et al., 2013), they cannot necessarily tell us what guides participants’ eye movements in more situated contexts such as spontaneous, multi-person interaction.

The second issue concerns the structure and analysis of turns at talk that have been used in third-person perspective eye-tracking studies. With the exception of some studies (Foulsham et al., 2010; Edlund et al., 2012; Hirvenkari et al., 2013), the stimuli in third-person perspective eye-tracking studies were scripted and strongly controlled, which has a range of implications. For one thing, it means that the dialogs were presumably fairly carefully spoken and had rather long gaps between turns and few if any overlaps. Indeed, in some cases the gaps between turns were 900 ms on average (Keitel et al., 2013), which considerably exceeds the ∼200 ms mean gap duration (Stivers et al., 2009) and the 0–200 ms mode of gap durations (Stivers et al., 2009; Heldner and Edlund, 2010) observed for spontaneous conversation. In fact, 900 ms gap durations are more representative of lengthened gaps marking dispreferred responses (Kendrick and Torreira, 2014). Careful pronunciation, lack of overlap, and relatively long gaps may, of course, all influence how turns are processed and projected.

Moreover, the structure of questions in spontaneous conversation is often complex, with more than one point of possible completion within a single turn. Conversation-analytic research on turn-taking has suggested that participants in conversation monitor turns for points at which they are recognizable as possibly complete because such points constitute opportunities for transition between speakers (Sacks et al., 1974). In the following example, the participant addressed by the question responds at the first point at which the question is possibly complete, even though the speaker of the question continues his or her turn, adding a term of address.

FD:IV:191 (Sacks et al., 1974, p. 702)

Desk: What is your last name [Loraine.

Caller:                                         [Dinnis.

Desk: What?

Caller: Dinnis.

Thus, the first possible completion of a question is not necessarily the end of the turn, as a speaker can continue to speak past this point. If participants in conversation do indeed monitor turns at talk for points of possible completion, as Sacks et al. (1974) proposed, then we may find evidence for this in the eye movements of unaddressed participants in question–response sequences. However, in studies using the third-person perspective paradigm, either the turns used as stimuli were constructed to have simple structures in which the first possible completion of the turn was coterminous with its end, or multiple possible completions were not taken into account in the analysis. As a consequence, it is currently unknown how the gaze behavior of observers is timed with respect to points of possible completion prior to the ends of turns as such. The literature on third-person perspective eye-tracking paradigms has referred to eye movements that precede the end of a turn as anticipatory. Since first possible completions are often not the end of the turn, gaze shifts that are anticipatory with respect to the end of the turn may actually follow a first possible completion point, or may virtually coincide with this point. The extent to which eye-movements do or do not anticipate the possible completion of a turn matters for the interpretation of results from this paradigm within models of turn-taking behavior, thus further underlining the need for a systematic consideration of the intricate structure of turns.

### THE PRESENT STUDY: INVESTIGATING EYE MOVEMENTS AND TURNS AT TALK *IN SITU*

The present study aims to shed light on the timing of eye movements and turns at talk by situating the third-person perspective eye-tracking paradigm within spontaneous, live conversations. To this end, using state-of-the-art technology, we studied a corpus of triadic conversations between friends and examined exchanges in which a speaker addressed a single participant, thus rendering the third a momentarily ‘unaddressed participant’ (Bolden, 2013; cf. ‘unaddressed recipient,’ Goffman, 1979, 1981; cf. ‘side-participant,’ Clark and Carlson, 1982; ‘audience,’ Levinson, 1988). More specifically, we tracked this person’s eye movements during question–response sequences to measure whether, and if so at precisely which point, unaddressed participants moved their eyes from current to next speaker. This approach builds on earlier work by moving from scripted dialogs involving actors to natural multi-person interaction in which participants experience personal immediacy and co-presence, the turns at talk are of direct relevance to them, and participants may become the addressee at any given moment. Moreover, the measurements of turns and gaps between them are not determined by the experimenter or actors but are natural in content and length. Further, we not only consider questions in their entirety but also apply a more fine-grained analysis, tackling the intricate structure of spontaneous questions by examining the timing of eye movements with respect to first possible completions, as well as the end of turns. Thus, we aim to answer not only the question of how eye movements are timed with respect to turns, but also to what extent they are governed by the projection of the current or next turn. Finally, while to date all reports have discussed observers’ gaze behavior across turn transitions in terms of the cognitive processes that underpin turn-taking, the present study also aims to consider the nature of this phenomenon as a social behavior. This will help us understand whether we are dealing with a turn-taking phenomenon *per se* or with one that belongs to some other order of conversational organization.

## MATERIALS AND METHODS

### PARTICIPANTS AND CORPUS

The corpus consists of ten groups of participants engaging in casual conversations in English recorded at the Max Planck Institute for Psycholinguistics in Nijmegen, The Netherlands. The recordings include both ten triadic (three participants) and ten dyadic (two of the three participants) conversations ^1^ (for an eye-tracking corpus of dyadic interactions in Flemish, see Brône and Oben, 2014). All conversations are ∼20 min. in length. For the eye-tracking analyses reported here, seven of the ten triadic conversations were analyzed as calibration was poor for the remaining three. All participants were native speakers of English recruited from the general Nijmegen population and knew each other prior to the recording session (except for one triad in which one person knew both of the other two participants who had not themselves met before). Their ages ranged from 19–68 years (Mean age = 30 year). Two of the conversations were all female, two all male, and three conversations consisted of two female and one male participant.

### LABORATORY SET-UP AND TECHNICAL EQUIPMENT

**FIGURE 1:**
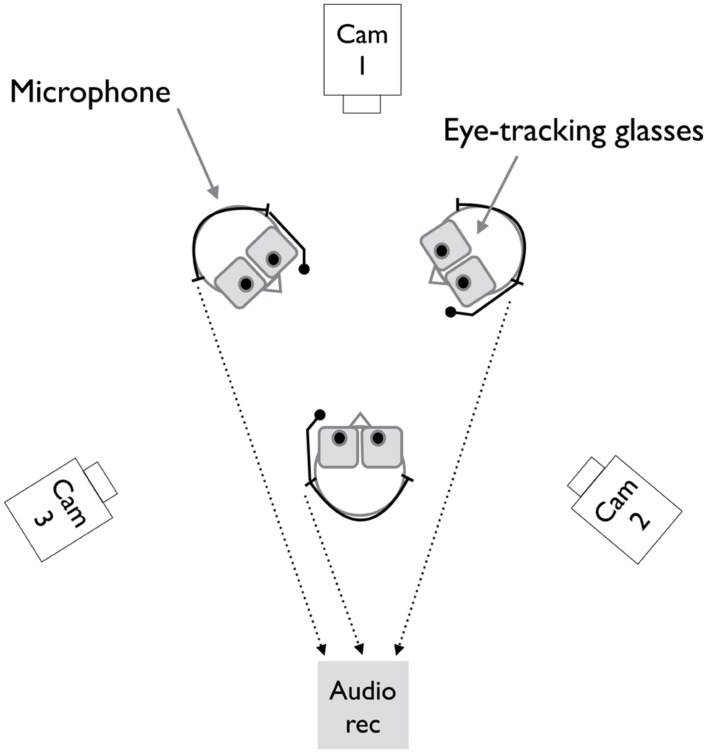
**Illustration of the technical laboratory set-up used in the present study**.

The recordings took place in a sound proof room equipped with professional lighting suitable for high quality audio and video recording. Participants sat in standard height chairs with armrests, arranged in a triangle with the chairs equidistantly placed from one another. A ceiling microphone recorded the entire conversation. Each participant wore a head-mounted lightweight uni-directional microphone (Shure SM10A), which recorded only the respective participant’s voice, and a pair of eye-tracking glasses (SMI, sampling rate 30 Hz). In addition, three HD video cameras (Canon Legria HFG10, 25 fps) recorded frontal views of each person (except for one triad where one of the three HD cameras failed to record; the respective participant’s data was not included in the present analysis). Due to the spatial arrangement of the chairs with respect to the cameras, each person was also visible from the right and left side in the recordings made by the respective other two video cameras. **Figure 1** provides an overview of the laboratory set-up and equipment.

**FIGURE 2:**
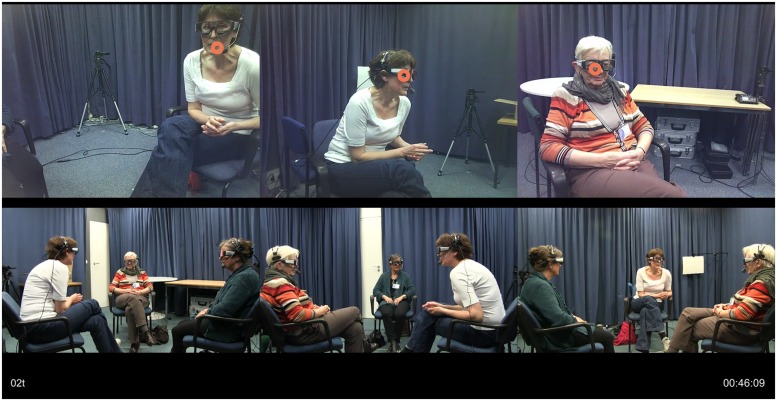
**Still frame from a synchronized six-video recording (one triad).** Top panel shows the three eye-tracker videos including gaze cursor (in orange); bottom panel shows the three HD camera recordings. The video of each participant’s view through the eye-tracker is positioned above the corresponding frontal HD video recording of this participant.

For each session, the recorded material resulted in three individual videos from the cameras, three individual videos from the eye-trackers (exported from the SMI recording device with the gaze cursor overlaid onto the visual scene recorded by the video cameras of the eye-trackers), three individual audio files, and the audio file from the ceiling microphone. The audio tracks were recorded in sync using a four-channel audio recorder (Edirol/Roland R-44). The six video recordings and three individual audio recordings were combined and synchronized in Adobe Premier Pro CS4 and then exported as a single audio–video file for analysis (MP4) at 24 frames per second (see **Figure 2**). This resulted in a time resolution of approximately 41 ms, the duration of a single frame. The synchronization was based on audible and visible information from a clapperboard used at the beginning and end of each session.

### PROCEDURE

Upon their arrival, participants were greeted by two investigators who conducted the study (JH and KK) and were handed study-packs, including information about the study and procedure of the session, forms asking about their language background, screening questionnaires ruling out motor and speech impairments, as well as consent forms and questionnaires about handedness and a variety of social dimensions. Once the study-packs had been completed by all participants (except for the social questionnaires, see below) and any queries had been answered, participants were seated in their chairs in the recording room. All equipment was prepared beforehand, allowing immediate fitting of the microphones and the eye-trackers (involving a three-point calibration procedure).

Each recording session lasted approximately 40 min. in total, with the first 20 min. constituting a trialogue phase and the second 20 min. a dialog phase. Upon completion of the initial fitting procedure, the two investigators left the room and waited in an adjacent area until the first 20 min. had elapsed. At this point, they compared performance of the three eye-trackers and asked the person wearing the eye-tracker with the poorest calibration to leave the room. Once the remaining two participants had talked for another 20 min., all three were reunited in the recording room and asked to complete the social questionnaires contained in the study-packs. This was to ensure that questions about human communication and behavior (verbal and non-verbal) would not influence participants’ behavior during the conversations. (The results from the social questionnaires are not of relevance for the present analysis and will not be discussed any further.) Participants were then asked one more time for their written informed consent relating to how their data should be handled, thanked, and financially compensated for their participation (26 euro per person). The entire test session lasted around 120 min. The study was approved by the Social Sciences Faculty Ethics Committee, Radboud University Nijmegen.

### ANALYSIS

#### Question-response sequences

The present analysis focused on question–response (henceforth QR) sequences in which the question was addressed to a single participant who then produced a response. All QR sequences were identified by an experienced conversation analyst (Kobin H. Kendrick), resulting in a total of 281 questions and their responses (a subset of which was included in the final gaze shift analysis, see Eye Gaze). Criteria for identifying QR sequences in our dataset were based on the coding scheme proposed by Stivers and Enfield (2010, pp. 2621–2626). The precise beginnings and endings of the questions and the responses were determined in Praat 5.3.77 (Boersma and Weenink, 2014). In-breaths preceding responses were clearly audible in our recordings and were treated as the onset of the response (*N* = 35). In a small number of cases (*N* = 2) the response was exclusively non-verbal (e.g., head nods); in those cases the beginning of the response was timed to the first frame of visible movement. These annotations were then imported into ELAN 4.61 (Wittenburg et al., 2006).

#### Points of possible completion

All questions in the dataset were analyzed for the presence and location of points of possible completions before the end of the turn, drawing on conversation-analytic research on turn construction (Sacks et al., 1974; Ford and Thompson, 1996; Ford et al., 2002). For a point of possible completion to be identified, the turn at talk up to that point must have been hearable to the analyst as a possibly complete question in its context. This determination was made holistically, with attention to the syntax, prosody, and meaning of the question. For those questions with a point of possible completion before the end of the turn, the precise location of the first possible completion was annotated in ELAN. Crucially, the participants’ gaze behavior was not considered in this analysis.

The analysis of points of possible completion revealed a number of recurrent types. If a turn contained two complete questions, a point of possible completion – represented here by a vertical bar – was identified after the completion of the first, whether the two questions were produced one after the other (e.g., “where does she go? — where- where does she- what uni’s she from?”) or with a short silence between the two (e.g., “but is it good? — (0.1) or is it just (0.2) any money is good?”). If a turn contained a possibly complete question together with an increment, a contingent addition to a turn that continues its grammatical structure (Schegloff, 1996; Couper-Kuhlen and Ono, 2007), a point of possible completion was identified before the increment (e.g., “how are you finding it by the way”; “were you on a bike — at that time?”). If the turn contained a tag question, a frequent occurrence in the dataset, a point of possible completion was identified before the tag (e.g., “there was like a fifth one — wasn’t there?; “you were at it too — right?”). And if the turn contained a possibly complete question followed by a turn component that could not have been projected or anticipated in advance, a point of possible completion was identified after the question (e.g., “all your family’s in England — I expect?”; “so it’s on campus this place?”).

Interrater agreement between two coders (KK and JH) who independently identified the presence and precise location of points of possible completion in the dataset revealed strong reliability, *K* = 0.72 (85.7% agreement; Landis and Koch, 1977).

#### Eye gaze

The ELAN files containing the QR annotations were linked to the synchronized videos in order to annotate the unaddressed participants’ eye movements during the QR sequences. These annotations were done manually, on a frame-by-frame basis. At each frame during the QR sequence, the gaze fixation point generated by the SMI software for the unaddressed participant (henceforth referred to as the gaze cursor) was categorized as being (1) on speaker A, (2) on speaker B, (3) on self (e.g., when looking at his or her own hands), (4) on the surroundings (e.g., the walls, the door, any equipment items in the room), or (5) as not identifiable from the eye-tracker data (i.e., the eye-tracker cursor was not visible in the respective video frames). Based on this coding scheme, 45 of the originally 281 QR sequences (16.0%) were discarded from further analysis of the unaddressed participants’ eye movements due to insufficient data. (Note that the eye movement data of unaddressed participants is associated with considerably more data loss than the eye movement data for speaker A and speaker B. This is because, in our set-up, unaddressed participants often move their heads as well as shift their gaze to look from the current to the next speaker, and these movements tended to be performed quite fast and with the eyes being closed during the shift, thus obscuring the corneal reflection the eye-tracker needs to capture).

Out of the remaining 235 QR sequences, unaddressed participants moved their gaze from speaker A to speaker B in 105 (45.5%) QR sequences. In order to be considered a valid gaze shift for our analysis, the trajectory had to be one that clearly moved from A to B, without the gaze pausing elsewhere in between (such as on self or background objects). In the remaining 131 sequences, unaddressed participants either did not shift their gaze at all and instead fixated speaker A, speaker B, themselves, or the surroundings throughout, or they did move their eyes but in the opposite direction, that is, from speaker B to speaker A. While these cases are interesting in themselves, they tap into a different phenomenon than the one under investigation here and require analysis and discussion elsewhere.

Regarding those 105 QR sequences that did reveal a gaze shift from speaker A to speaker B (i.e., our final QR dataset), the average question duration was 2018 ms (Median = 1681 ms; minimum value = 328 ms; maximum value = 7667 ms), and the average response duration was 1899 ms (Median = 1312 ms; minimum value = 164 ms; maximum value = 8118 ms). Due to the highly dynamic nature of conversation brought about by, amongst other things, differences in personality, age, gender, closeness of friendship, and topic of discussion, the seven triads of course differed in the number of QR sequences they contributed to our analysis (they contributed 2, 8, 10, 12, 16, 22, and 35 QR sequences, respectively). Likewise, participants within the triads differed in the extent to which they contributed to the conversation by asking questions, but none of the conversations excluded participants (and those that asked fewer questions may, of course, have contributed more to the conversation in other ways, such as through tellings, jokes, responses, and so forth). Basing analyses of QR sequences in conversation on samples that are determined by the participants’ spontaneous behavior, thus resulting in varying numbers of QR sequences across separate interactions, is the standard procedure for corpus studies and in line with existing research (e.g., Stivers et al., 2009, 2010; Gardner, 2010; Strömbergsson et al., 2013).

**FIGURE 3:**
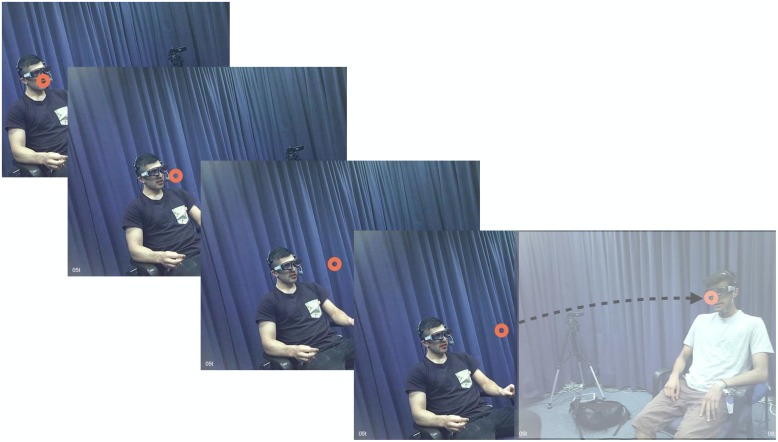
**Consecutive still images providing an example of an unaddressed participant’s gaze shifting away from speaker A toward speaker B during a QR sequence.** Frames 1 and 2 capture instances of the gaze cursor being classed as on speaker A (see coding criteria), whereas frame 3 captures the first gaze shift away from speaker A (moving to speaker B, frame 4).

For these 105 QR sequences that did reveal a shift of the unaddressed participant’s gaze from speaker A to speaker B, we identified *when* exactly this gaze shift occurred. The time window we took into consideration for identifying gaze shifts relevant for this analysis stretched from the beginning of A’s turn to the end of B’s turn. In all cases of gaze shifting from speaker A to speaker B within this time window, unaddressed participants looked at the face of speaker A and then moved their gaze from there to the face of speaker B. Using the frame-by-frame gaze annotations, we identified the first frame at which the gaze cursor left speaker A, defined as the frame at which the gaze cursor was no longer on, overlapping with, or directly adjacent to speaker A’s head or technical head-gear (see **Figure 3**). At what time point before or during B’s turn the unaddressed participant’s gaze arrived at speaker B was not of relevance for the present analysis. Annotations were made in ELAN to measure the duration from the first gaze shift away from speaker A by the unaddressed participant to two points within the question turn: (1) the end of the turn and (2) the first point of possible completion of the question, for those questions that had a possible completion before the end of the turn. In addition to measuring the duration of these intervals (in ms), the values were set as either positive or negative. This was done to identify the temporal order of the respective events, with negative values indicating an anticipatory gaze shift before a point of possible completion or the end of a turn and positive values indicating the inverse.

However, we need to consider that it takes time to plan and launch these eye movements before they are observable. This process is estimated to take on average around 200 ms (Salthouse and Ellis, 1980; Fischer and Ramsperger, 1984; Becker, 1991; Allopenna et al., 1998; Griffin and Bock, 2000). We therefore calculated a value for the beginning of the assumed planning phase for each observed value by subtracting 200 ms.

Gaze coding was performed by two independent coders (LD and MvdG) blind to the study’s predictions and assumptions. In addition, their coding was checked by one of the two senior analysts (JH and KK), and any errors in coding (of which there were remarkably few due to the clear categorical distinctions between gaze locations) were discussed and corrected. Due to the considerably more objective coding criteria applied for our gaze analysis in comparison to the identification of points of possible completion, formal reliabilities were calculated for the latter only.

#### Statistical analysis

All statistical analyses were conducted in R 3.1.1 (R Core Team, 2012). The density plots displayed in the Results section were generated using the Lattice package (Sarkar, 2008) with default kernel density estimation (Gaussian). Since these distributions render a smoothed curve (rather than a histogram) and an *estimate* of the mode, all mode values given should be considered close approximations of the true value and decimal places are not stated for those values. Note also that these distributions are based on binned data brought about by our video frame rate providing a measure every 41.7 ms (24 fps).

## RESULTS

Out of the 105 QR sequences analyzed here, 54.3% (*N* = 57) of the questions had at least one possible completion before the end of the turn. Here, we focus our analyses first on the end of the turn and then on its first possible completion (which corresponded to the end of the turn for 45.7% of questions).

### EYE MOVEMENTS TIMED WITH RESPECT TO THE END OF TURNS

**FIGURE 4:**
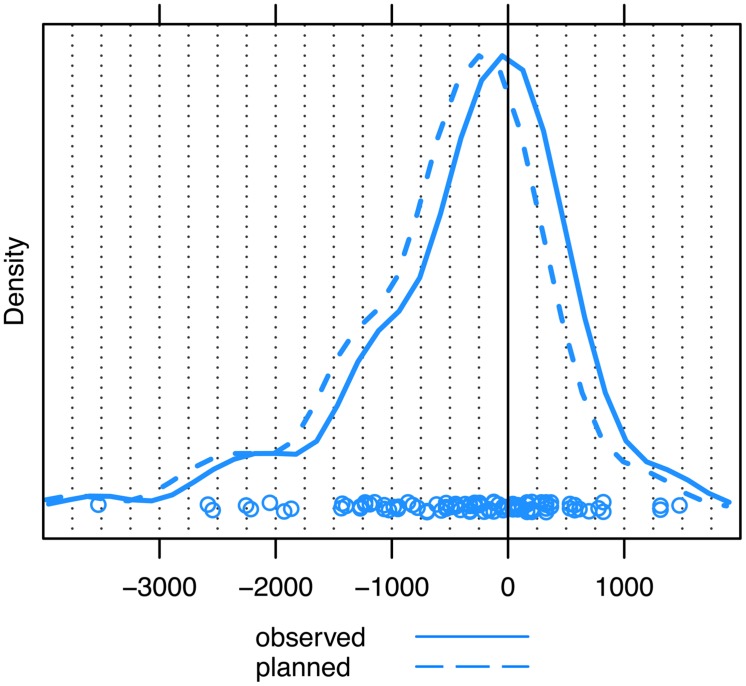
**Unaddressed participants’ first gaze shift away from speaker A to speaker B, measured with respect to the end of the question (solid line = observed eye movements, dashed line = planned eye movements).** The zero point on the *x*-axis (ms) marks the end of the question turn. The peak of the distribution represents the estimate of the mode. Dots represent the individual datapoints.

First, we measured the time point of each first observed gaze shift away from speaker A (and toward speaker B) with respect to the end of speaker A’s turn. This showed that the estimate of the mode of these data is located very close to the end of the question, namely just 50 ms prior to turn end (see **Figure 4**, solid line). Because, as we have already noted, experimental research indicates that overt eye movements are planned about 200 ms in advance of them being observed, the covert initiation of unaddressed participants’ eye movements from speaker A to speaker B occurred most frequently around 250 ms prior to the end of questions (see **Figure 4**, dashed line).

On the whole, 60.0% (*N* = 63) of QR sequences were associated with observable gaze shifts that anticipated the end of the question turn. When taking into account the time it takes to prepare these gaze shifts, the percentage of anticipatory gaze shifts increases to 73.3% (*N* = 77).

### EYE MOVEMENTS TIMED WITH RESPECT TO THE FIRST POSSIBLE COMPLETION

**FIGURE 5:**
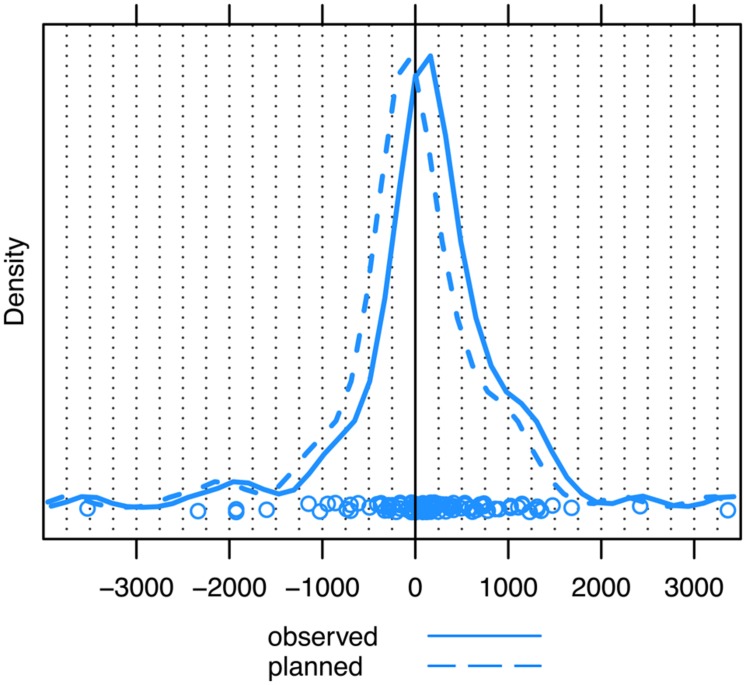
**Unaddressed participants’ first gaze shift away from speaker A to speaker B, measured with respect to the first possible completion of the question (solid line = observed eye movements, dashed line = planned eye movements).** The zero point on the *x*-axis (ms) marks the first possible completion of the turn. The peak of the distribution represents the estimate of the mode. Dots represent the individual datapoints.

Because many of the questions in our data had a point of possible completion prior to turn end (as seen above), we carried out a second analysis in which we timed unaddressed participants’ first gaze shift away from speaker A with respect to the first possible completion of each question; this corresponded to the end of the turn for those questions with only one possible completion. When plotting our data with respect to this reference point, the distribution yields a mode of about 160 ms just after the first possible completion (see **Figure 5**, solid line). Taking into account the 200 ms required to plan and launch observed eye movements, the distribution yields a mode of 40 ms just prior to the first possible completion (see **Figure 5**, dashed line).

When timing the gaze shifts with respect to the first possible completion of the question, we still see that a considerable number of gaze shifts from current to next speaker happen prior to the first possible completion, but much less so than when timing these gaze shifts with respect to the end of the turn: in 34.3% (*N* = 36) of cases, unaddressed participants shifted their gaze from current to next speaker before the first possible completion, and in 55.2% (*N* = 58) of cases unaddressed participants’ gaze shifts had at least been planned prior to this point.

### UNADDRESSED PARTICIPANTS’ EYE MOVEMENTS AND ADDRESSED PARTICIPANTS’ RESPONSES

Above we have shown that unaddressed participants are sensitive to first possible completions, as can be seen from the timing of their gaze shifts. However, considering that first possible completions mark points at which transition to the next speaker becomes relevant, addressed participants, too, are likely to be sensitive to these points and time their responses to them. This means that the first possible completion of speaker A’s question and the onset of speaker B’s response may often coincide. We therefore also measured the timing of the response ^2^ to see whether its onset may have attracted unaddressed recipients’ attention and thus account for the timing of the gaze shifts we observed. And indeed, when we tested this statistically on our data, the result yielded a significant correlation between the unaddressed participants’ first gaze shift from speaker A to speaker B and onset of speaker B’s response [ρ(13) = 0.234, *p* < 0.05). This means that for responses that coincide with first possible completions of questions, gaze shifts could either be due to unaddressed participants recognizing the possible completion or reacting to the onset of the response. In order to tease these two factors apart, we carried out two further analyses by looking at two subsets of our data.

**FIGURE 6:**
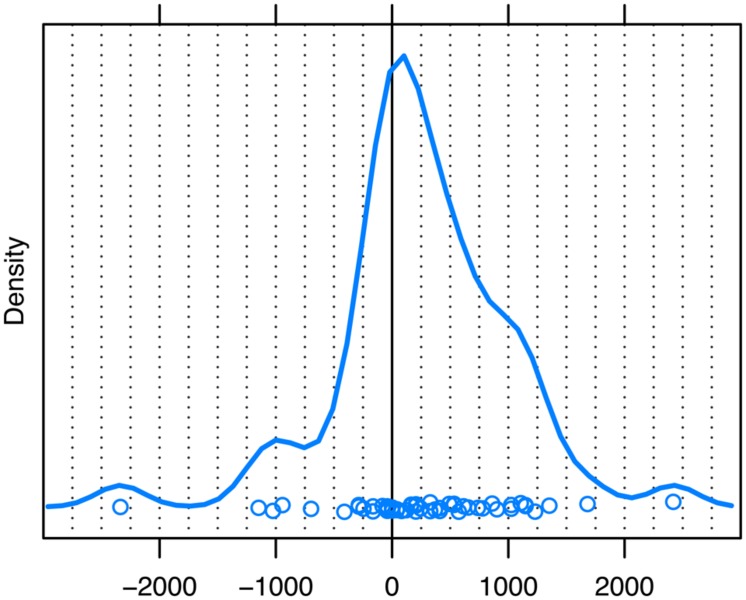
**Unaddressed participants’ first gaze shift away from speaker A to speaker B for responses with an onset of 200 ms or more *after* the first possible completion of the question.** The zero point on the *x*-axis (ms) marks the first possible completion of the turn. The peak of the distribution represents the estimate of the mode. Dots represent the individual datapoints.

For the first analysis, we considered only those QR sequences where speaker A’s first possible completion and speaker B’s response onset did not coincide but where the response comes after the possible completion. For this comparison, we selected those sequences where the response occurred more than 200 ms ^3^ after the first possible completion (*N* = 54). If the timing of the unaddressed participants’ gaze shifts we observed based on the sample as a whole is explained by response onset rather than by first possible completions, then the mode for this subset of data should be at least 200 ms later than the mode for the distribution based on the entire sample. However, as can be gleaned from **Figure 6**, the mode for this subset is 105 ms (Range = –2337–2419), which differs only slightly from the mode of 160 ms for the entire sample. If anything, unaddressed participants’ observed gaze shifts occur slightly earlier when B’s response occurs 200 ms after the first possible completion, and certainly no later than when we consider the entire sample. Thus, unaddressed participants’ eye movements in our data do indeed appear to reflect sensitivity to the first possible completion of the question, rather than being a mere reaction to the onset of the response.

**FIGURE 7:**
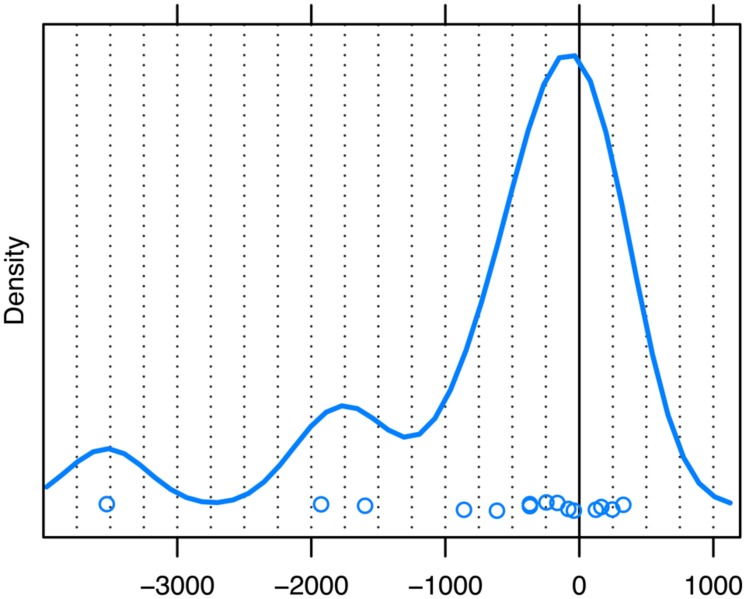
**Unaddressed participants’ first gaze shift away from speaker A to speaker B for responses with an onset of 200 ms or more *prior* to the first possible completion of the question.** The zero point on the *x*-axis (ms) marks the first possible completion of the turn. The peak of the distribution represents the estimate of the mode. Dots represent the individual datapoints.

However, we of course do acknowledge that response onset may also play a role in the timing of unaddressed participants’ eye movements. In order to explore this further, we looked at another subset of our data, namely those cases in which speaker B’s response began at least 200 ms before speaker A’s first possible completion (*N* = 15). If response onset alone also attracts unaddressed participants’ attention and, as a consequence, their gaze, then we should see that the mode of the distribution of gaze shifts for this subset is earlier than that for the distribution based on the sample as a whole. As can be seen from **Figure 7**, this was indeed the case, with the mode of observed gaze shifts for the subset of early responses being -35 ms, compared to an overall mode of 160 ms. This means that the eye movements within this subset must have been planned -235 ms before the first possible completion, which corresponds closely to the onset of these early responses at -200 ms or less.

## DISCUSSION

The present study sought to investigate the precise timing of unaddressed participants’ eye movements during question–response sequences by advancing on previous research in two important ways. Firstly, we immersed the third person within the situated context of a spontaneous, live conversation in which he or she was a ratified participant (Goffman, 1979, 1981). Secondly, we aimed to zoom further into the pattern of anticipation established in previous studies by taking into account the intricate structure of questions in conversation. In addition, we here consider whether unaddressed participants’ eye movements during question–response sequences are a turn-taking phenomenon *per se*, or whether they reflect processes of a different conversational order.

### UNADDRESSED PARTICIPANTS TRACK CURRENT SPEAKERS

First of all, our analyses show that even when unaddressed participants are directly immersed in a conversation (rather than being third-person observers of pre-recorded dialogs), they move their gaze from one speaker to the next in about half of all question–response sequences. This confirms that, even when participating in actual ‘on-line’ interaction, unaddressed participants show a tendency to track current speakers (cf., von Hofsten et al., 2009; Foulsham et al., 2010; Tice and Henetz, 2011; Casillas and Frank, 2012, 2013; Edlund et al., 2012; Hirvenkari et al., 2013; Keitel et al., 2013), at least during QR sequences.

### UNADDRESSED PARTICIPANTS SHIFT THEIR GAZE BEFORE TURN ENDS

When we examined the timing of gaze shifts with regard to turn ends, we found that the most frequent gaze shifts from current to next speaker were planned around 250 ms and observable around 50 ms prior to turn end. Thus, the results suggest that unaddressed participants’ gaze shifts are predominantly anticipatory in nature when timed with respect to the end of question turns. As such, it replicates the findings from third-person perspective eye-tracking studies that have found evidence for adults shifting their gaze to the next speaker prior to turn end (e.g., Tice and Henetz, 2011; Casillas and Frank, 2012). Overall, this suggests that the third-person perspective eye-tracking paradigm, at least when used with adults and in the context of question–response sequences, serves as a good experimental proxy for investigating the eye movements of unaddressed participants in the context of turn-taking. A valuable follow-up to the present study may be one that employs the video recordings filmed from the unaddressed participants’ view in a third-person perspective eye-tracking study as this would allow for a more direct comparison with the situated behavior to further corroborate this point.

However, despite the comparability, gaze shifts prior to turn end appear to be more common in actual conversation. In the present study, the majority of gaze shifts from current to next speaker occurred prior to turn end (60% of cases) or were planned and launched prior to this point (73% of cases). In contrast, in studies using the third-person perspective paradigm, either only a relatively small proportion of gaze shifts occurred prior to turn end (e.g., Tice and Henetz, 2011; Casillas and Frank, 2012) or none at all did (e.g., Edlund et al., 2012; Hirvenkari et al., 2013). Keitel et al. (2013) found that 54% of their adults’ gaze shifts were anticipatory in nature – a proportion much closer to the present findings – but this percentage includes all gaze shifts made between 500 ms prior to the end of the current turn, as well as all gaze shifts made during the on average 900 ms gap preceding the next turn. It is therefore not possible to evaluate the extent to which these gaze shifts were anticipatory with respect to the end of the current turn, the measure we applied in the present study. In all, while gaze shifts do appear to be more anticipatory in actual conversation than in off-line eye-tracking paradigms, we have to consider that the present study focused exclusively on question–response sequences rather than on a mixture of different turn types. Since Casillas and Frank (2012) found a trend toward slightly stronger anticipation for questions than for non-questions for adults, we have to be mindful that this may also explain, or at least contribute, to the stronger pattern of anticipation found in the present study.

### UNADDRESSED PARTICIPANTS SHIFT THEIR GAZE AT POSSIBLE TURN COMPLETIONS

The present study went further than just measuring eye movements with respect to turn ends. Here, we have taken into account the intricate structure of questions, and, more specifically, the first possible completion of each question, which for half of our questions was not the actual end of the turn. These points of possible completion create opportunities for a next speaker to take the turn, and it has been argued that participants in conversation are sensitive to these transition-relevance places (Sacks et al., 1974). Indeed, our data seem to corroborate this: we found that, in the majority of cases, unaddressed participants initiated the planning of their gaze shifts most frequently just 40 ms prior to the first possible completion of the turn. This time interval is shorter than the average duration of a single vowel in English (House, 1961; Umeda, 1975) and suggests that the planning of the most frequent gaze shifts more or less coincides with the point in the current turn at which transition between speakers first becomes relevant. Indeed, our measurement of the location of possible completions within a turn, which identifies them at the end of a word, is conservative. If the possible completion becomes recognizable even earlier, for example, as the result of an increase in the duration of final words or segments (see Local et al., 1986; Gravano and Hirschberg, 2011), the initiation of planning (i.e., the peak of the distribution in **Figure 5**) would occur after the possible completion, not before.

Thus, rather than a pattern of anticipation, in which unaddressed participants project the ends of question turns in advance, the virtual coincidence of possible completions and the onset of planning suggests that unaddressed participants recognize points of possible completion as they occur. That is, they seem to perceive specific cues closely associated with, and thus indicative of, the emergence of possible completions. Wells and Macfarlane (1998) have argued that transition relevance places can be defined in prosodic terms and that specific final major accents of a current turn signal its upcoming completion (cf. Schegloff, 1996, on ‘pitch peaks’ as indications of possible completion). They conclude that next speakers need not anticipate this accent; they merely have to recognize it. However, even the recognition of final accents or pitch peaks is a process that unfolds over time. The observation that gaze shifts are planned and launched 40 ms before the first possible completion of the current turn could therefore be interpreted as projection on a micro-scale, as it were, but it is something quite different from the long-range projection that has been argued for by some. Schegloff (1987) has proposed that the initial components of a turn can facilitate the projection of how it will end, *well before* it reaches possible completion (see also Levinson, 2013). This means that the grammatical structure of questions would allow unaddressed participants to shift their gaze to the next speaker at a very early point during the question. Considering that addressees are non-verbally responsive as speakers’ utterances unfold (Clark and Krych, 2004; Bavelas and Gerwing, 2011; Traum et al., 2012), unaddressed participants may well feel inclined to gaze at the next speaker as early as possible to see how the emerging utterance is received. However, the present findings suggest that early projection of this kind does not govern the eye movements of unaddressed participants as they redirect their gaze from current to next speakers in question–response sequences. We do concede that unaddressed participants are likely to engage in sequence projection processes from very early on, which tells participants *what* is coming next (a response; Schegloff, 2007), and thus where to move their eyes (to the respective next speaker). However, local cues associated with the emergence of possible completion, rather than early turn projection, appear to act as a launch-signal by telling participants *when* to move their eyes.

Crucially, we have also shown that first possible completions govern unaddressed participants’ gaze shifts in the absence of an early response. First possible completions alone appear to account for much of the data in our sample. (For 68.7% of our QR sequences the onset of the response came after the first possible completion.) At the same time, however, we have been able to show that early responses which precede the first possible completion also attract unaddressed participants’ gaze, and thus may certainly be a contributing factor in those instances where first possible completion and onset of the response coincide. Hence, taking overlap between current and next speakers into account appears crucial if we aim to understand unaddressed participants’ eye movements in natural conversation. Importantly, the effect of both factors – first possible completions and response onset – is based on a process of *recognition* rather than projection.

### OPTIMIZING RECIPIENCY

To date, all studies of the phenomenon under investigation here – the redirection of gaze by unaddressed participants from current to next speakers at turn transitions – have used it to gain insight into processes involved in turn-taking. But the conclusion that the eye movements of unaddressed participants do not anticipate the first possible completion of the current turn, and thus do not necessarily reflect a projection of it, leads us to reconsider the nature of the phenomenon and to look elsewhere for principles that can account for the fine temporal coordination that we observe.

It has long been argued that among the many functions of gaze behavior in social interaction the use of gaze to display attention, engagement in the interaction, and recipiency to the current speaker is paramount (Goodwin, 1980, 1981; Heath, 1984, 1986; Kidwell, 1997; Robinson, 1998; Ruusuvuori, 2001; Ford and Stickle, 2012). Gazing at the current speaker not only shows one to be an attentive participant, whether directly addressed by the turn or not, but it also allows one to tap into the rich stream of visible behaviors that accompany turns at talk. Our results reveal that unaddressed participants redirect their gaze at a moment that is interactionally most optimal: by moving their eyes away from the current speaker not at the beginning of the question but close to its completion, unaddressed participants secure access to as much of the current speaker’s visible bodily behavior as possible, including torso, head, and hand gestures, as well as lip movements and facial expressions that accompany the communicative action; at the same time, they also secure access to much of the next speaker’s visual response to the question. Further, keeping their gaze on the current speaker until a very late point during the question allows unaddressed participants to display recipiency throughout most of the question, just as the reorientation to the addressed participant at the completion of the question allows them to do for the response. Both of these aspects, the visual behavior of speakers and its temporal coordination with possible turn completions, as well as the use of gaze for displaying and managing recipiency in multi-person interaction, are currently being investigated in more detail. This will help us to unravel the specific ways in which these factors contribute to the processing of turns and the organization of gaze in social interaction.

Although the gaze behavior of unaddressed participants does not necessarily reflect projection of the current turn, optimizing recipiency between current and next speakers does make use of the turn-taking system in other ways. Our results provide new and quantitative evidence that the recognition of points of possible completions are indeed core to the turn-taking system in conversation, as argued in Sacks et al.’s (1974) seminal paper. Moreover, it appears that not only addressed but also momentarily unaddressed participants orient to possible completions as they process turns at talk. This observation further underscores the point by Sacks et al. (1974, p. 727) that the organization of turn-taking creates an “intrinsic motivation for listening.” One who wishes to have a turn at talk must attend to and process the current turn in order to recognize a point at which transition between speakers may occur. Even unaddressed participants, who do not take a turn in the question–response sequences in our data, show evidence in their gaze behavior of a fine attunement to this feature of the turn-taking system ^4^. Our findings that unaddressed participants’ gaze behavior during question–response sequences appears to be organized according to a principle that optimizes recipiency also fits well with the notion of an ‘intrinsic motivation for participation,’ as it were (Schilbach et al., 2010; Pfeiffer et al., 2014). Both Schilbach et al. (2010) and Pfeiffer et al. (2014) demonstrate that, in the context of gaze-based interactions, humans experience social-interactional engagement as rewarding, as evidenced by cerebral activity patterns in reward-related neurocircuitry.

In addition, it appears from our results that response onset can trump first possible completions, at least when these responses come prior to the first possible completion. In such cases, the timing of the response appears marked and may signal a marked social action (see Vatanen, 2014). That unaddressed participants orient their gaze toward the participant issuing a response of this status, despite the current turn not yet having reached its first possible completion, neatly fits the principle of optimizing recipiency.

The present study looked at eye movements with respect to one particular type of turn, that is, questions. Casillas and Frank (2012) found a marginally significant effect indicating that, in third-person perspective paradigms, adults show a stronger tendency to shift their gaze from current to next speaker – and a trend for this happening slightly earlier – for questions than non-questions. Corpus research on the timing of turn-taking in spontaneous conversation, however, found that participants responded as quickly to questions as to non-questions (Stivers et al., 2009). Further research on different types of turns is thus clearly needed and may help to explain why analyses that have combined questions with other turn types have not found evidence of anticipatory eye movements (Hirvenkari et al., 2013). Moreover, the present study focused on those question–response sequences that were associated with patterns of gaze behavior which would allow us to draw conclusions about unaddressed participants’ cognitive processes relating to the anticipation of turn ends and upcoming responses. Question–response sequences associated with different gaze patterns (such as unaddressed participants continuing to gaze at the questioner throughout the entire sequence) are not informative in this respect. Note that we are not suggesting that entirely different cognitive processes are at work in those cases. Quite the opposite – while it is very likely that unaddressed participants recognize possible turn completions also during those kinds of question–response sequences, other processes appear to be governing their eye movements causing them not to shift their gaze toward the responder at this point. What exactly these processes are is an open question and certainly worthy of future research, but they address a different question to the one under investigation here.

## CONCLUSION

The present study has provided us with a first glimpse of the intricate connections between turns at talk and unaddressed participants’ eye movements in spontaneous, multi-person interaction. On the one hand, we have here reproduced the basic findings from studies using the third-person perspective eye-tracking paradigm in spontaneous, live conversation. On the other, our data have provided us with stronger evidence that gaze shifts by unaddressed participants toward next speakers precede the end of the current turn than previous studies have. As such, our findings corroborate the notion that interactive paradigms do, at least in part, provide different insights than paradigms involving passive observation (Schilbach, 2010, 2014; Wilms et al., 2010; Pfeiffer et al., 2013; Schilbach et al., 2013). Further, the present study has advanced our understanding of which structures in the current turn guide unaddressed participants’ eye movements in conversation and has helped to clarify the role that the projection of the current turn plays in this process. While our findings underline the general usefulness of third-person paradigms, they also point toward some of the limitations associated with this approach. Moreover, they point to the urgent need to consider not just actual turn ends but also first possible turn completions when measuring and interpreting eye movements during turns at talk. Finally, the present study has allowed us to identify a new interactional phenomenon, the optimization of recipiency, which appears to account for much of the gaze behavior of unaddressed participants during turn-taking.

## Conflict of Interest Statement

The authors declare that the research was conducted in the absence of any commercial or financial relationships that could be construed as a potential conflict of interest.

## Footnotes

1 Due to the present focus being on unaddressed participants’ eye movements, the dyads are reported here for completeness sake but are not analyzed in the present study.

2 This measure considers vocalization as well as inbreaths produced in preparation for the response proper since preparatory inbreaths serve as pre-beginnings of turns (Schegloff, 1996) and thereby signal an intention to speak. Gaps and overlaps (i.e., gaps with negative values) were thus measured as the duration from the end of speaker’s A turn to the beginning of speaker B’s response, including audible inbreaths (see Materials and Methods). For sequences in which questions had only one possible completion (i.e., the first possible completion equated to the end of the turn), responses most frequently occurred after a gap of approximately 120 ms (Mode est. = 120 ms; Range = -943–1968 ms), with 31.3% (*N*=15) of responses in overlap with the question. For sequences in which questions had more than one possible completion (i.e., the first possible completion was not the end of the turn), the picture was predictably more complex, as responses could begin with reference to either of the two points. The distribution of gap durations in such sequences was non-unimodal, with a plateau between two slight peaks at -500 and 20 ms, and with 64.9% (*N*=37) of responses in overlap with the question.

3 An earlier time point, such as 100 ms after the first possible completion, would also have been a viable comparison, but we settled for 200 ms since the hypothesized difference in modes would be clearer, and because the time needed to plan and execute an observable gaze shift would by that point have elapsed.

4 Note that, while our analysis suggests a close coordination of eye gaze and turn structure, this does not exclude the possibility that participants’ gaze is not also organized with respect to the structure of larger sequences and courses of actions (Rossano, 2012).
